# Lentiviral transduction facilitates RNA interference in the nematode parasite *Nippostrongylus brasiliensis*

**DOI:** 10.1371/journal.ppat.1009286

**Published:** 2021-01-26

**Authors:** Jana Hagen, Peter Sarkies, Murray E. Selkirk

**Affiliations:** 1 Department of Life Sciences, Imperial College London, London, United Kingdom; 2 MRC London Institute of Medical Sciences, Imperial College London, London, United Kingdom; University of California Riverside, UNITED STATES

## Abstract

Animal-parasitic nematodes have thus far been largely refractory to genetic manipulation, and methods employed to effect RNA interference (RNAi) have been ineffective or inconsistent in most cases. We describe here a new approach for genetic manipulation of *Nippostrongylus brasiliensis*, a widely used laboratory model of gastrointestinal nematode infection. *N*. *brasiliensis* was successfully transduced with Vesicular Stomatitis Virus glycoprotein G (VSV-G)-pseudotyped lentivirus. The virus was taken up via the nematode intestine, RNA reverse transcribed into proviral DNA, and transgene transcripts produced stably in infective larvae, which resulted in expression of the reporter protein mCherry. Improved transgene expression was achieved by incorporating the *C*. *elegans hlh11* promoter and the *tbb2* 3´-UTR into viral constructs. MicroRNA-adapted short hairpin RNAs delivered in this manner were processed correctly and resulted in partial knockdown of β-tubulin isotype-1 (*tbb-iso-1*) and secreted acetylcholinesterase B (*ache-B*). The system was further refined by lentiviral delivery of double stranded RNAs, which acted as a trigger for RNAi following processing and generation of 22G-RNAs. Virus-encoded sequences were detectable in F1 eggs and third stage larvae, demonstrating that proviral DNA entered the germline and was heritable. Lentiviral transduction thus provides a new means for genetic manipulation of parasitic nematodes, including gene silencing and expression of exogenous genes.

## Author summary

The complex life cycle of parasitic nematodes makes them very difficult to manipulate genetically, and methods to delete or silence genes which are routinely used in other organisms are ineffective in most species of nematodes which infect animals. This has hindered attempts to understand the function of defined genes and proteins, and their roles in development and interaction of nematode parasites with their host. We show here that foreign genetic material can be introduced into a widely used laboratory model of intestinal nematode infection by using a viral vector. The vector was modified to improve transgene expression, and a reporter protein expressed by transduced nematode larvae in vitro. We subsequently utilised the viral vector to deliver double stranded RNA molecules to the larvae. These molecules were processed along known pathways, resulting in partial knockdown of two test genes. This system represents a new means of genetically manipulating nematode parasites, and will aid in understanding their complex biology, in addition to defining new targets for control of infection.

## Introduction

Nematode parasites are responsible for infection of large numbers of people and animals, and the latter issue represents a global agricultural problem which restricts economic growth in many countries [1]. No subunit vaccines for nematode infection have been commercialised, and multi-drug resistance to the major classes of anthelmintics is widespread in gastrointestinal parasites of livestock [2]. Definition of new chemotherapeutic targets is hampered by the lack of methods for gene silencing to define critical points of intervention. Development of robust silencing protocols would also allow the identification of proteins that are essential for suppression of host immunity and parasite survival, which in turn would facilitate vaccine development.

Although RNA interference in animals was discovered and characterised in *Caenorhabditis elegans* [3], it has proven extremely difficult to translate to animal-parasitic nematodes, i.e. those which infect humans, livestock and laboratory animal models. Methods employed thus far have used soaking, electroporation and bacterial feeding with double stranded (ds) and small interfering (si) RNAs, with inconsistent results across different species, tissues and targets [4–6]. Systematic comparison indicates that most of the RNAi machinery is conserved across parasitic nematodes, with notable exception of the *sid-2* gene, which encodes an intestinal luminal transmembrane protein required for environmental RNAi in *C*. *elegans* [7,8]. The major blockade to RNAi in most parasitic nematodes is thus likely to be effective delivery of sufficient dsRNA to trigger the silencing mechanism.

To solve the problem of delivery of the RNAi trigger, we investigated whether parasitic nematodes could be transduced with lentivirus, an approach which has been successfully employed to deliver microRNA-adapted short hairpin RNAs (shRNAmirs) and co-opt the miRNA pathway to achieve gene knockdown in *Schistosoma mansoni* [9]. Viral transduction also introduces the possibility of delivering hairpin dsRNAs for RNAi [10], or other applications associated with transgenesis in general. We chose to examine *Nippostrongylus brasiliensis*, as it is a widely used laboratory model of gastrointestinal nematode infection which has been particularly important in defining mechanisms of host immunity [11]. It is RNAi-competent [12] although currently with very inefficient and irreproducible results [13]. Recent data indicate that, contrary to previous thought, the parasite feeds on blood from infective larvae (L3s) through to adult stages, and it is thus a good lab model for haematophagous nematodes such as hookworms [14].

## Results

### Lentiviral transduction of activated L3s

**Fig 1:**
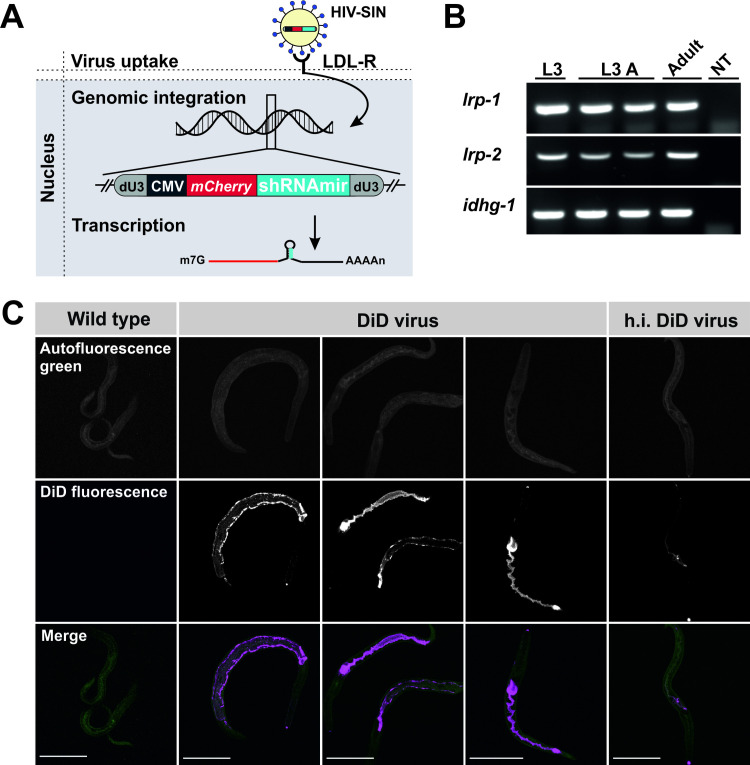
Lentiviral transduction of activated L3s. (A) Schematic of lentiviral transduction of cells for delivery of a mCherry expression cassette under the control of the CMV promoter following binding of viral particles to the LDL receptor. A microRNA-adapted short hairpin (shRNAmir) targeting the gene of interest is encoded in the 3´-UTR of mCherry transcripts. (B) Detection of LDL receptor orthologues (*lrp-1* and *lrp-2*) by RT-PCR from different stages of *N*. *brasiliensis*. *Idhg-1* (Isocitrate dehydrogenase subunit) was amplified to control for cDNA integrity. L3A, activated L3s; NT, no template control. C) Uptake of DiD-labeled lentiviral particles. Activated L3s were exposed to DiD-labeled lentiviral particles (DiD-virus) or left untreated (wild type, WT) and analysed for fluorescence by confocal microscopy after 24 h. A heat–inactivated virus control (h.i. DiD-virus) was included. Fluorescence was detected at the intestinal epithelium of larvae exposed to viable DiD-labeled lentiviral particles alone. Autofluorescence in the green channel (blue laser) served as counterstain. Scale bar: 100 μm.

In a first series of experiments we assessed whether third stage larvae (L3s) could be transduced with VSV-G-pseudotyped lentiviral particles for delivery of a mCherry expression cassette under the control of the CMV promoter (Fig 1A). Notably, L3s and adult worms of *N*. *brasiliensis* express orthologues of low-density lipoprotein receptor (LDLR)-related (LRP) proteins [15] (Fig 1B), the receptor family utilised by VSV-G to enter mammalian host cells [16]. Prior to exposure to lentiviral particles, *N*. *brasiliensis* L3s were activated by culture at 37°C in the presence of rat serum for 48–72 hrs. The temperature shift acts as a cue for exsheathment and induces feeding in L3 [17]. Activated larvae were incubated with lentivirus labelled with 1,1-Dioctadecyl-3,3,3,3-tetramethylindodicarbocyanine (DiD), and subsequent imaging by confocal microscopy showed the virus localised to the parasite intestinal epithelium, most likely following fusion of the viral envelope with the host cell membrane (Fig 1C).

**Fig 2:**
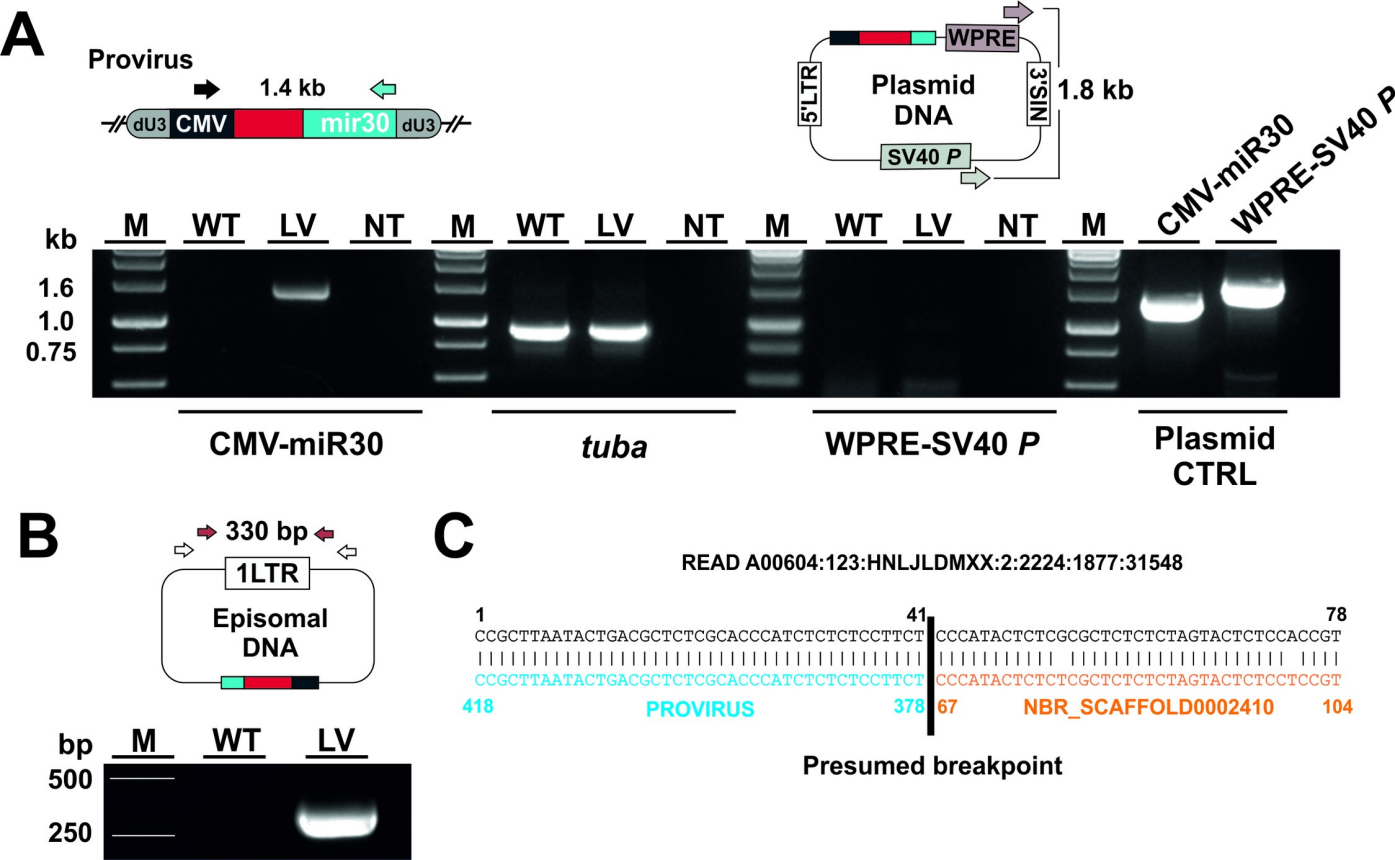
Detection of proviral DNA in transduced L3s. Genomic DNA was isolated from lentivirus-exposed (LV) or unexposed (wild type, WT) L3s after three days and analysed for proviral DNA by PCR. (A) The presence of proviral DNA in worm gDNA was confirmed by PCR of the transgene expression cassette resulting in a 1.4 kb amplicon (CMV-miR30). *Tubulin alpha chain* (*tuba)* was amplified to control for gDNA integrity. The absence of residual plasmid DNA in gDNA preparations was confirmed by PCR with primers binding the WPRE and SV40P region (WPRE-SV40 *P*). Amplification of lentivirus-encoding plasmid DNA served as a positive control (Plasmid CTRL). NT, no template control. (B) Detection of episomal circular 1-LTR viral DNA by Nested PCR confirms successful reverse transcription of viral genomic RNA in transduced worms. The location of primer pairs used for nested PCR step one (white arrows) and two (red arrows) is indicated. (C) Example of viral integration site. Split read showing alignment with lentiviral DNA and *N*. *brasiliensis* genomic DNA.

Three days after exposure to lentiviral particles, the transgene expression cassette was detected by PCR of worm genomic DNA resulting in a 1.4 kb fragment (Fig 2A), providing evidence for successful reverse transcription of viral RNA into proviral DNA. The absence of PCR fragments amplifying the WPRE and SV40 promoter region in transduced L3s indicated that amplification of the expression cassette was not due to residual plasmid DNA in genomic DNA preparations. Moreover, the detection of episomal circular one-long terminal repeat (1-LTR) viral DNA further confirmed successful delivery and reverse transcription of viral RNA in *N*. *brasiliensis* L3s (Fig 2B).

Reverse transcription of viral RNA indicates successful transport into the nucleus. However, upon entering the nucleus, the pre-integration complex can integrate as provirus into host cell genomic DNA or remain in an episomal state, such that PCR does not provide proof of genomic integration. We therefore investigated this by whole genome sequencing. As expected, significantly more reads aligning to viral DNA were observed in genomic DNA of worms exposed to virus than unexposed worms (S1A Fig). Clear evidence of viral integration was provided by split reads that aligned partly to viral DNA and partly to the *N*. *brasiliensis* genome (Fig 2C). We detected such events at a rate of 2 per 30 million reads (S1B Fig).

### Functionality of the expression cassette and viability of transduced worms

**Fig 3:**
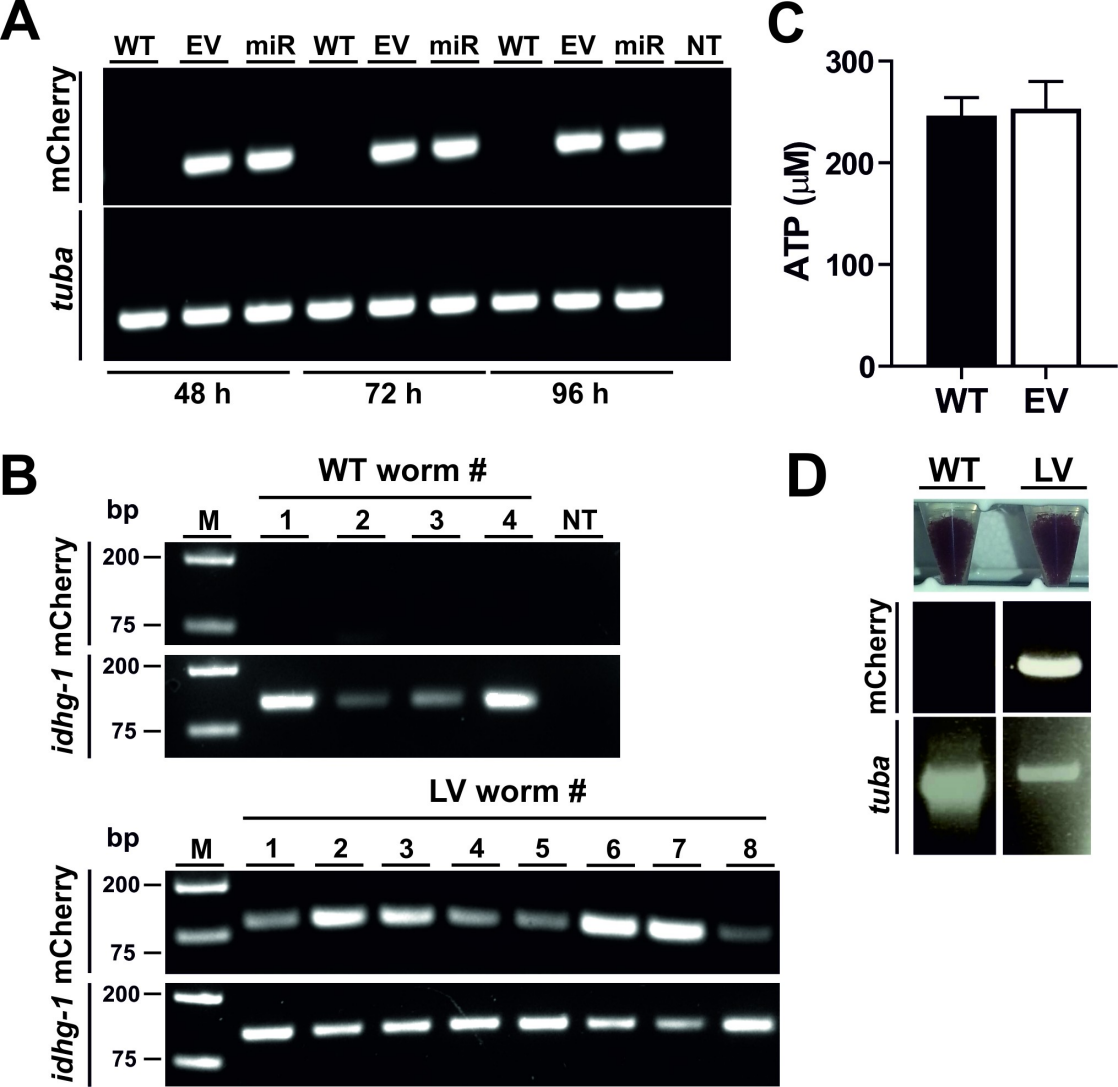
Functionality of the expression cassette and viability of transduced L3s. (A) Detection of mCherry transcripts over a time course of 96 hrs post-exposure to lentivirus encoding mCherry under the control of the CMV promoter. Activated L3s were exposed to lentivirus with (miR) or without (EV, ‘empty vector’) a complete shRNAmir structure or left untreated (wild type, WT) and analysed for presence of the transgene in cDNA. *Tuba* (tubulin alpha chain) was amplified to control for DNA integrity. NT, no template control. (B) Single worm RT-PCR to detect proportion of larvae expressing mCherry. Individual L3s either transduced with lentivirus (LV worm) or unexposed (WT worm) were used to amplify virus-encoded mCherry or control parasite *idhg-1* transcripts as described in Materials and Methods. (C). Viability of L3 larvae was assessed by ATP assay 72 hrs after transduction. Bars represent the mean ± SEM of ATP content for three biological replicates (100 worms per sample). (D) Recovery of transduced worms from infected host animals. Activated L3s were exposed to lentivirus (LV) for 6 hrs or left untreated (wild type, WT). Worms were washed extensively before infection of host animals (rats). Adult worms were recovered from the intestines 6 days post-infection and analysed for presence of the mCherry transgene by PCR of genomic DNA.

We next assessed functionality of the expression cassette. Following transduction of *N*. *brasiliensis* L3s, transcripts of mCherry under the control of the CMV promoter were detectable when assayed for up to four days in vitro (Fig 3A). In order to determine the proportion of the L3 population that were transduced with virus and expressing mCherry, we performed single worm RT-PCR. Virus-encoded mCherry was detected in all larvae exposed to lentivirus (Fig 3B), and this was verified in a repeat experiment with the same number of worms. Unexposed larvae were negative for mCherry amplicons, and all larvae were positive for amplification of cDNA corresponding to the parasite *idhg-1* gene (Fig 3B). Importantly, transduction did not impair the viability of L3s as determined by ATP assay (Fig 3C) or affect their infectivity and development, as similar numbers of adult worms were recovered from intestines of infected host animals (Fig 3D). Transduced adult worms recovered from the intestines tested positive for the mCherry transgene by PCR of genomic DNA confirming stable transduction (Fig 3D). However, mCherry transcripts were not detected in adult worms recovered from infected animals in three independent experiments, suggesting that the lentivirus integration site or expression cassette is subjected to epigenetic silencing during parasite development.

### Proviral DNA is heritable

**Fig 4:**
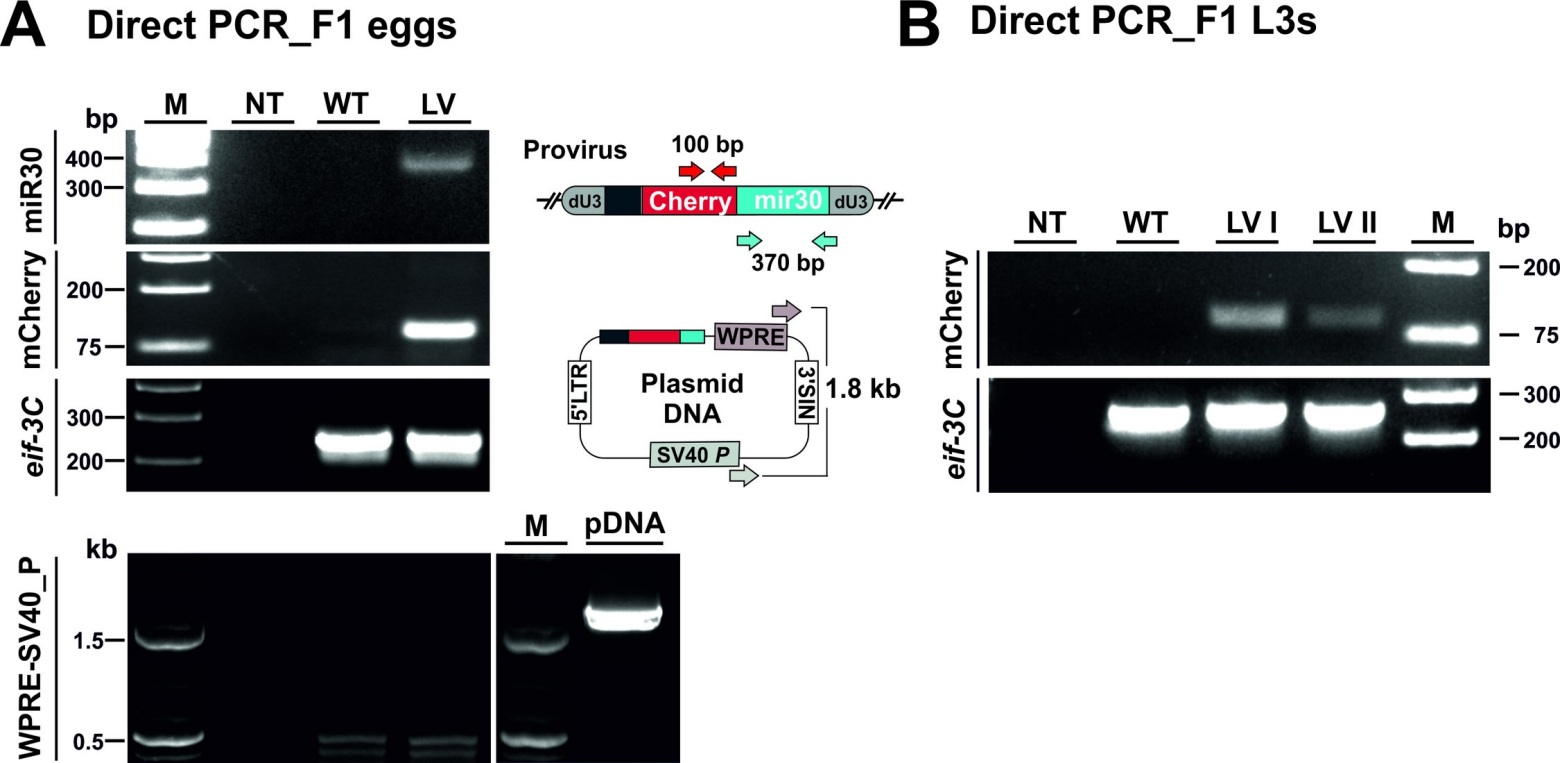
Detection of proviral DNA in the F1 generation. Eggs and L3s were recovered from the F1 generation following infection of rats with larvae transduced with or unexposed to lentivirus. (A) The presence of proviral DNA in eggs was confirmed by PCR of mCherry and miR-30, detected in F1 eggs following infection with transduced parasites (LV) but not those unexposed to virus (WT). Amplification of *N*. *brasiliensis eif-3C* was confirmed in all samples to control for gDNA integrity (*eif-3C*). The absence of residual plasmid DNA was confirmed by PCR with primers to the WPRE and SV40P region (WPRE-SV40). Amplification of lentivirus-encoding plasmid DNA (pDNA) served as a positive control. NT, no template control. (B) The presence of proviral DNA in L3s was confirmed by PCR of mCherry, detected in F1 L3s following infection with parasites transduced with lentivirus but not those unexposed to virus (WT). LVI and LVII represent F1 generation L3s derived from infection of 2 different rats with transduced parasites. Amplification of *N*. *brasiliensis eif-3C* was confirmed in all samples to control for gDNA integrity. NT, no template control. Approximately 2,000 L3s or eggs were used for PCR reactions following the single worm PCR protocol described in Materials and Methods.

We next questioned whether integrated proviral DNA was heritable. Rats were infected with transduced L3s and faeces collected on day 7 and day 8 post-infection for recovery of F1 L3s from faecal cultures. Adult worms were recovered at day 8 post-infection and cultured overnight in vitro. F1 eggs recovered from culture medium were assessed for the presence of proviral sequences by PCR. Two distinct virus-encoded sequences (mCherry and miR-30) were amplified from F1 eggs, but were absent from eggs recovered from rats infected with untransduced worms (Fig 4A). The absence of a WPRE-SV40 PCR fragment again confirmed that detection of mCherry and miR-30 sequences was not due to residual plasmid DNA (Fig 4A). Presence of provirus in the F1 generation was further confirmed by amplification of mCherry from L3s recovered from faecal cultures (Fig 4B). These experiments clearly demonstrated that lentiviral sequences entered the germline and were heritable in the next generation, although as with adult worms, mCherry transcripts were not detected. However, while virus-encoded sequences were detected in bulk preparations of eggs and L3s (approximately 2,000 in either case), we failed to detect mCherry by PCR on 16 single L3s, despite strong signals from *eif-3C* control reactions.

### Optimisation of the expression cassette

**Fig 5:**
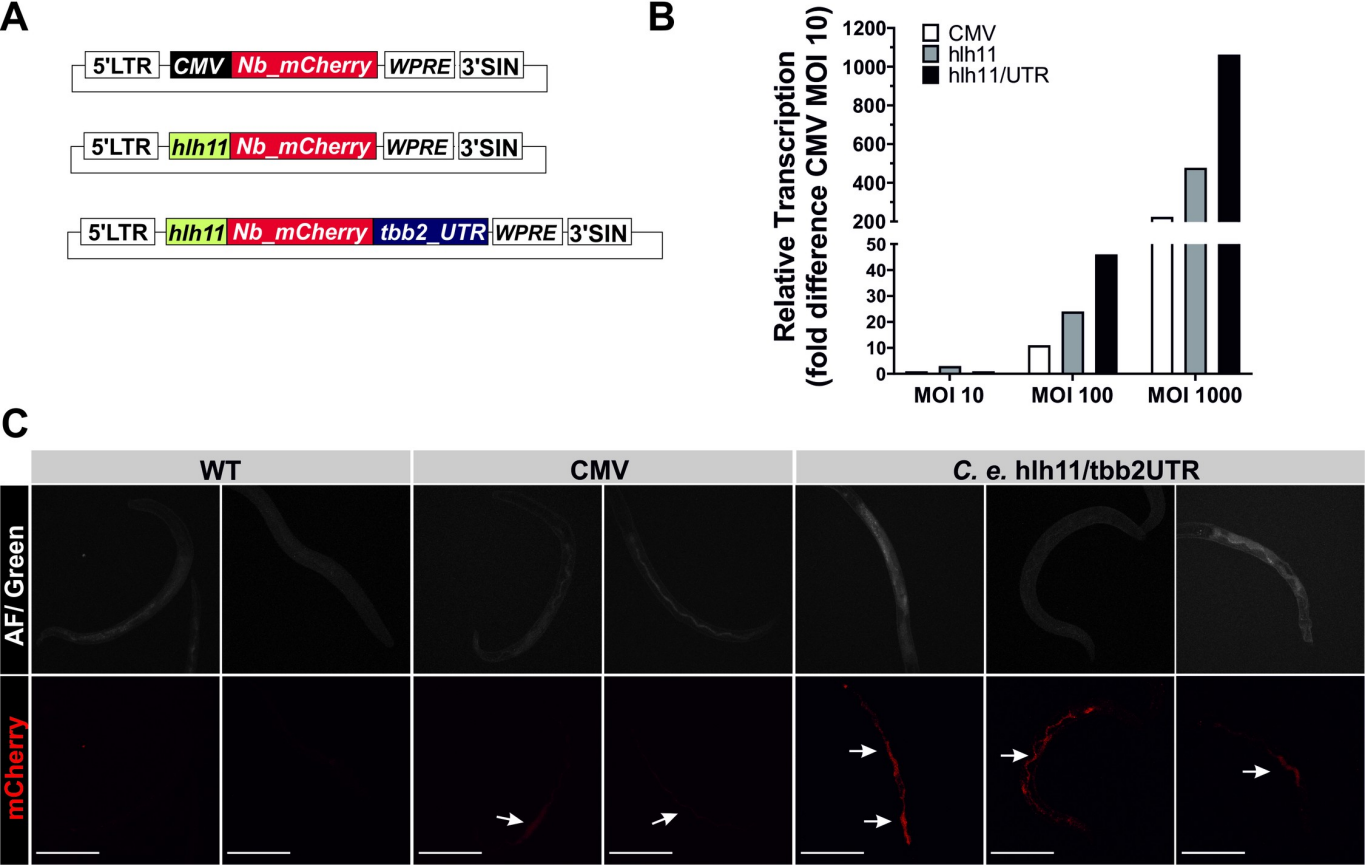
*C. elegans*-derived promoter and 3´-UTR enhances transgene expression in transduced L3s. Activated L3s were exposed to lentivirus encoding mCherry under the control of different promoter constructs and analysed for transgene expression 72 hrs later. (A) Schematic of the three expression cassettes tested. The mCherry sequence was optimised for *N*. *brasiliensis* codon usage (Nb_mCherry) and expression placed under the control of the CMV promoter or the *C*. *elegans*-derived hlh11 promoter in conjunction with the 3´-UTR of the *C*. *elegans tbb-2* gene. (B) Relative transcription of mCherry under the control of the different promoter constructs at different multiplicity of infection (MOI) was assessed by qPCR relative to the CMV promoter construct at a MOI of 10. Transcription was normalised to the geometric mean of Ct values for *rbd-1* and *idhg-1*. Data are from a representative experiment with 1,000 worms per sample group. (C). Worms were transduced at a MOI of 400 and analysed after 72 hrs for red fluorescence by confocal imaging. Autofluorescence in the green channel (blue laser) served as counterstain. Arrows indicate localisation of mCherry in transduced worms. Scale bar: 100 μm.

Given the apparent silencing of mCherry transcription during parasite development, we attempted to optimise the expression cassette by incorporation of 5´ and 3´ regulatory elements. The most active promoters in the *C*. *elegans* genome (so-called “hot” regions) are located within CpG-rich regions enriched for the H3K4me3 modification [18]. These regions bear similarity to mammalian CpG islands, which have been successfully used to improve lentiviral delivery [19]. We therefore cloned the *hlh11* “hot” region [18] and 3´-UTR of the β tubulin gene *tbb-2* from *C*. *elegans* [20] into our shRNA delivery vector (Fig 5A), and the mCherry reporter sequence was optimised for *Nippostrongylus* codon usage (Nb-mCherry) to improve translation.

The ability of different 5´ and 3´ regulatory sequences to drive transcription of the Nb-mCherry reporter was assayed by RT-qPCR, using serially increasing multiplicity of infection (MOI) of *N*. *brasiliensis* L3s with lentivirus. No difference in performance of these constructs was detected at a MOI of 10. However, substitution of the CMV promoter with *hlh11* resulted in a 2-fold increase of Nb-mCherry transcripts at MOIs of 100 and 1000, and addition of the *tbb2* 3´-UTR further improved transcription levels to 5-fold of that driven by the CMV promoter at higher MOIs (Fig 5B). Analysis of reporter expression by confocal microscopy showed that the *hlh11*/*tbb2*-UTR construct resulted in detectable Nb-mCherry fluorescence in L3s three days after exposure to lentivirus (Fig 5C). Despite enhanced transcription with this new construct in L3s, mCherry transcripts were still not detectable in adult worms by RT-PCR in two independent experiments. Nevertheless, improvements to the expression cassette made here provided the basis for studies to evaluate gene knockdown in transduced parasites.

### Transcriptional knockdown of *beta-tubulin-1* and *-2* in L3s following transduction with dsRNA constructs

**Fig 6:**
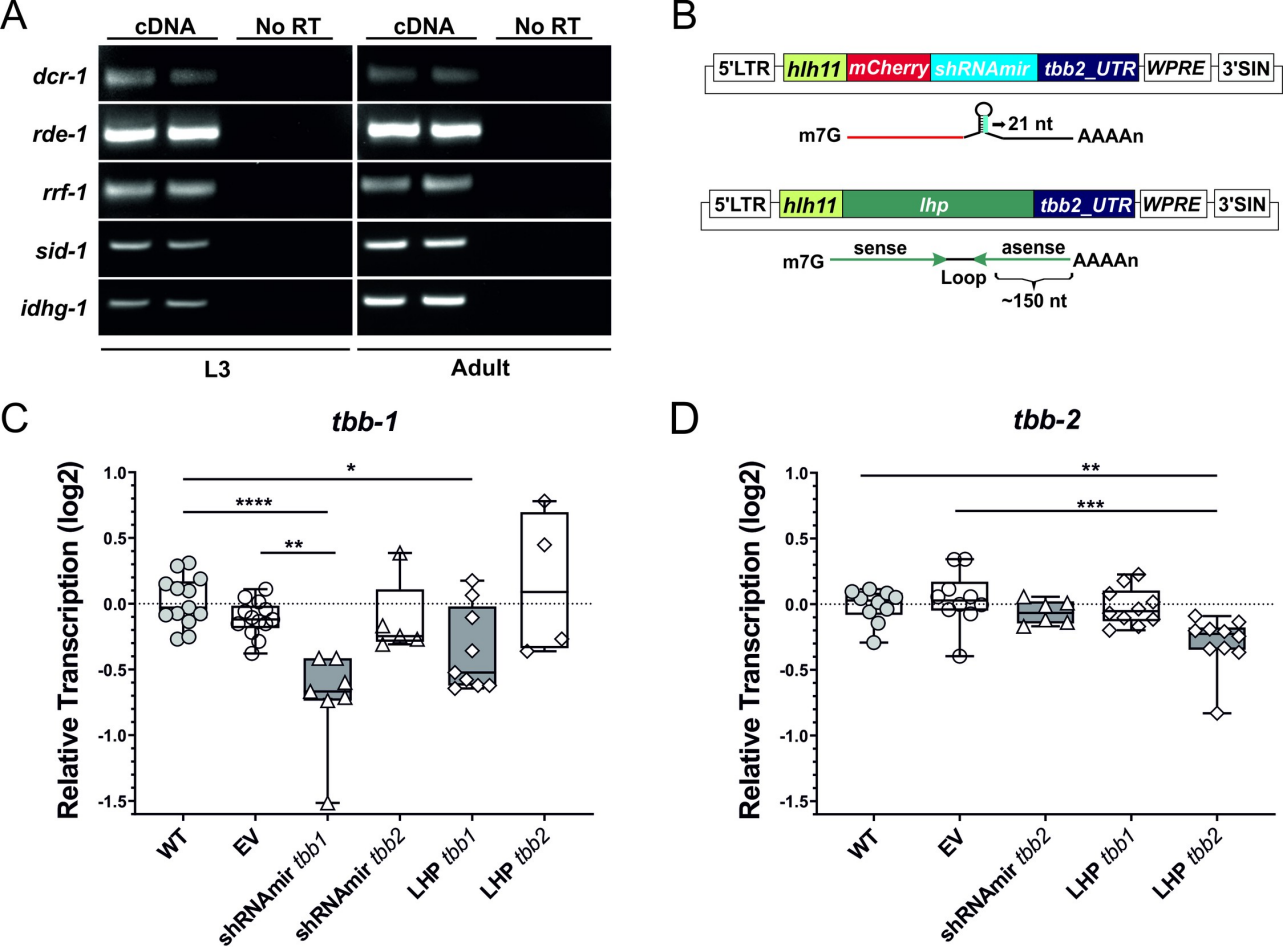
**Transcriptional knockdown of *beta-tubulin* 1 and 2 in L3s following transduction with dsRNA constructs. (**A) Detection of *dcr-1*, *rde-1*, *rrf-1* and *sid-1* orthologue transcripts in two different samples of activated L3s and adult worms of *N*. *brasiliensis* by RT-PCR. Amplification of *idhg-1* confirms cDNA integrity. NT, no template control; NoRT, no reverse transcription control. (B) Schematic of lentiviral expression cassettes encoding a target gene-specific shRNAmir placed immediately downstream of the mCherry coding region and upstream of the tbb2 3´-UTR, or a ~400 nt long hairpin structure (lhp) consisting of a 186 or 145 bp stem and 64 or 55 nt loop sequence (*tbb-1* or *tbb-2*, respectively). (C) Down-regulation of *tbb-1* or (D) *tbb-2* transcription in activated L3s three days after transduction. L3s were exposed to a cocktail consisting of four shRNAmir lentiviruses targeting either *tbb-1* or *tbb-2* (Table 1) or with lentivirus encoding a lhp targeting *tbb-1* or *tbb-2* transcripts. Control worms were left untreated (wild type, WT) or transduced with virus encoding mCherry lacking a hairpin sequence (empty vector, EV). Transcription of *tbb-1* or *tbb-2* in L3s was assessed by RT- qPCR three days after transduction relative to the wild type control worms and normalised against the geometric mean of Ct values of reference genes *eif-3C*, *idhg-1* and *rbd-1*. Box plot representing the median and upper/lower quartile of a data pool of 4 (*tbb-1*) or 3 (*tbb-2*) independent experiments with 3–4 biological replicates consisting of ~2,000 worms each. Whiskers indicate the highest or lowest value. Treatment groups were analysed for significant differences with the Kruskal-Wallis test and Dunns *post-hoc* test in relation to the wild type or empty vector control group. Statistical significance: ns = not significant, p<0.05 (*), p<0.01 (**), p<0.001 (***), p<0.0001 (****).

To establish proof of principle, we tested the ability of two different hairpin designs to induce transcriptional knockdown of the β tubulin genes *tbb-1* and *tbb-*2 in *N*. *brasiliensis* activated L3s. The first construct contained a shRNAmir, whereas the second contained a long hairpin (lhp) structure consisting of a 186 or 145 bp stem and 64 or 55 nt loop sequence, and these were constructed for both *tbb-1* or *tbb-2* (Fig 6B). The aim of the shRNAmir was to exploit the highly conserved miRNA pathway to achieve gene knockdown, whereas a lhp should generate several different primary siRNAs against the target, potentially leading to the production of secondary siRNAs known as 22G-RNAs in *C*. *elegans*, predominantly 23G-RNAs in *N*. *brasiliensis* [21], which might enable more potent knockdown [22]. Both L3s and adult worms express orthologues of components of the siRNA pathway required for 22G-RNA generation in *C*. *elegans*, and a *sid-1* orthologue, which may allow for transport of primary siRNAs throughout the worm (Fig 6A). To evaluate which method was more effective in inducing mRNA knockdown, we cloned four siRNA regions predicted within the lhp targets as shRNAmir 21 nt stems (Table 1), and worms were exposed to a cocktail of the four resulting lentiviruses targeting either *tbb-1* or *tbb*-2.

**Table 1:** Mature shRNAmir sequences targeting *tbb-1*, *tbb-2* or *ache-b* transcripts. The number indicates the position of the first nucleotide of the target sequence.

shRNAmir	Sequence 5´-3´
**tbb1-174**	AUUGAUUCUUUCCAGCUGCAG
**tbb1-180**	AUAGACAUUGAUUCUUUCCAG
**tbb1-185**	UUAUAAUAGACAUUGAUUCUU
**tbb1-283**	AUAACUGUCCAUAUGGUCCGG
**tbb2-130**	AUAGACAUUGAUUCGCUCCAA
**tbb2-135**	UUGUAAUAGACAUUGAUUCGC
**tbb2-246**	UUGUCCGGUCGGAACAGCUGG
**tbb2-444**	AUCUUUGCAAUCAACAACGUU
**aceb-468**	UUGACAUUCACAACUACUGCG
**aceb-1653**	UUGUCUUCGAUCUUUCUCGUU

Transduction with the shRNAmir-tbb1 cocktail resulted in a 40% (mean log2 ± SEM = -0.72 ± 0.14) knockdown of *tbb-1* transcripts three days after exposure to virus compared to wild type control worms (Fig 6C). No significant differences in transcription levels were observed with the empty vector virus (log2 = -0.11 ± 0.04), and importantly the shRNAmir-tbb-2 cocktail (log2 = -0.12 ± 0.13), indicating specificity of knockdown. Similarly, a 20–30% (log2 = -0.36 ± 0.11) decrease of *tbb-1* transcripts was recorded following the delivery of a lhp targeting *tbb-1* transcripts, but not with a lhp targeting *tbb-2* transcripts (log2 = 0.15 ± 0.28), further indicating target gene specificity of the delivery system (Fig 6C). Interestingly, a ~20% (log2 = -0.29 ± 0.07) reduction of *tbb-2* transcripts was detected following delivery of a lhp targeting *tbb-2* transcripts, while no reduction of transcription levels (log2 = -0.06 ± 0.04) was observed following delivery of a shRNAmir-tbb2 expression cassette (Fig 6D).

### Transcriptional knockdown of *secreted ache-B* in L3s following transduction with dsRNA constructs results in generation of 22G-RNAs

Although *tbb-1* and *tbb-2* are broadly expressed in many tissues of *C*. *elegans*, the susceptibility of *Haemonchus contortus tbb-1* to knockdown is thought to be due to expression in the intestine [23]. We therefore assessed whether transcripts expressed specifically in tissues other than the intestine could be targeted using lentiviral delivery. We chose *ache-b*, which encodes a secreted acetylcholinesterase expressed in subventral excretory glands [24], previously subjected to RNAi using conventional techniques with inconsistent results [13].

**Fig 7:**
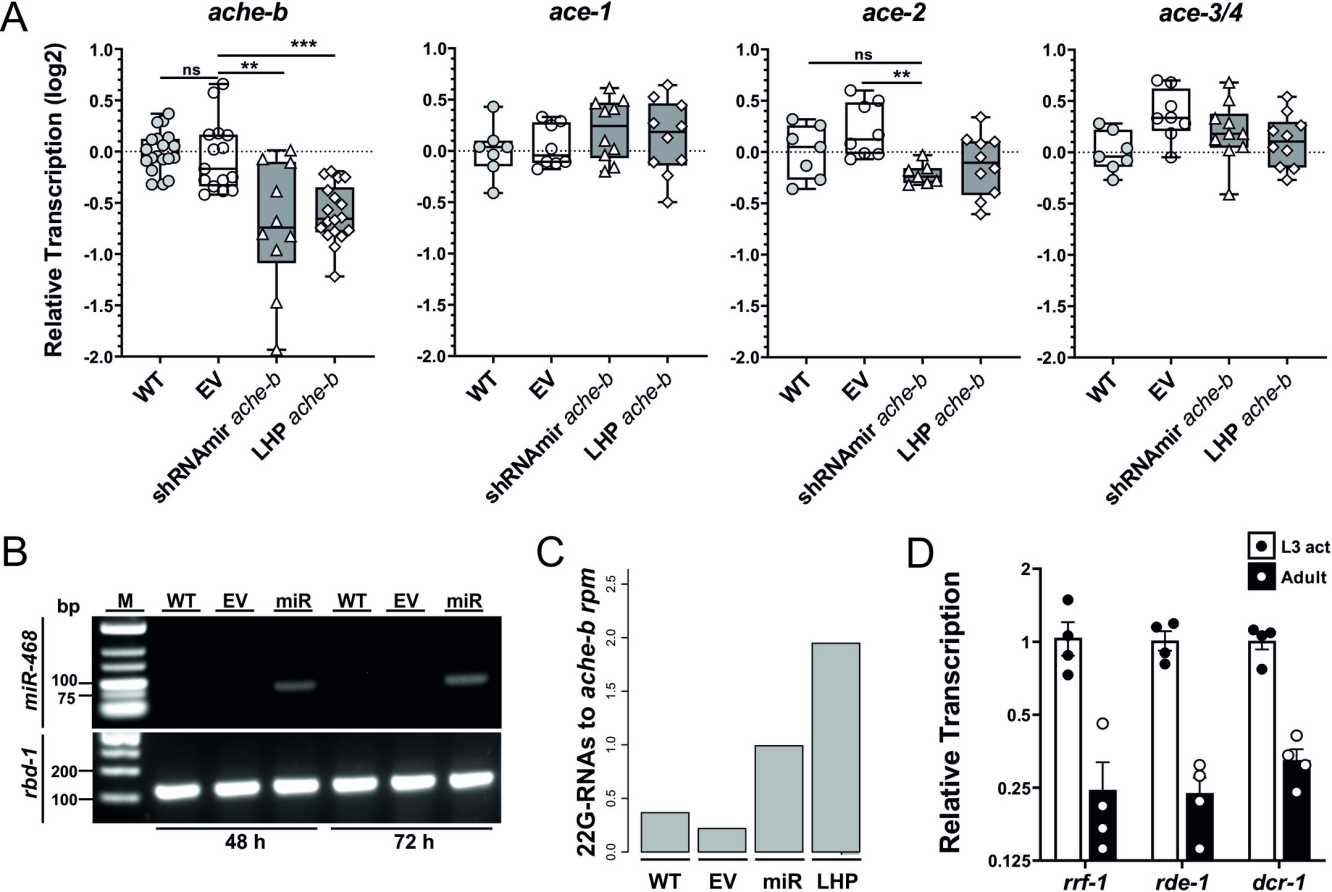
Transcriptional knockdown of secreted *acetylcholinesterase B* and processing of dsRNA in L3s following transduction with dsRNA constructs. (A) Down-regulation of secreted *ache-b* transcripts in activated L3s three days after transduction. L3s were exposed to a cocktail consisting of two shRNAmir lentiviruses (Table 1) or with lentivirus encoding a 250 bp lhp targeting *ache-b*. Control worms were left untreated (wild type, WT) or transduced with virus encoding mCherry lacking a hairpin sequence (empty vector, EV). L3 transcripts were assessed by RT-qPCR three days after transduction relative to wild type control worms and normalised against the geometric mean of Ct values of reference genes *eif-3C* and *idhg-1*. Box plot representing the median and upper/lower quartile of a data pool of 6 (*ache-b*) or 3 (*ace-1*,*2*,*3/4*) independent experiments with 2–4 biological replicates consisting of ~2,000 worms each. Whiskers indicate the highest or lowest value. Treatment groups were analysed for significant differences with the Kruskal-Wallis test and Dunns *post-hoc* test in relation to the wild type or empty vector control group. Statistical significance: p<0.05 (*), p<0.01 (**), p<0.001 (***) (B) Detection of mature miRNA in transduced worms. Total RNA was isolated from transduced worms at 48 or 72h post exposure to virus and analysed for presence of mature miRNAs generated from shRNAmir_468 using miScript detection system (Qiagen) and miRNA specific forward primer, resulting in a ~85 bp amplicon. (C) Detection of secondary siRNAs (22-G RNAs) generated from dsRNA in adult worms. L3 worms were transduced with lentivirus and introduced into host animals to complete their life cycle. Adult worms were recovered 5 days post infection. Small RNA libraries were prepared from total RNA and analysed following deep sequencing. The y axis shows 22G-RNAs to *ache-b* reads per million (rpm) total small RNAs. (D) Quantitation of transcripts for components of the exogenous RNAi pathway in activated L3s and adult worms was assessed by RT-qPCR. Interleaved scatter plot with bars and mean ± SEM of 4 biological replicates.

Transduction of activated L3s with a cocktail consisting of 2 viruses encoding different shRNAmirs (*acheb*-468 and *acheb*-1653) or a 250 bp lhp construct resulted in a ~40–50% (mean log2 ± SEM = -0.72 ± 0.2 and -0.61 ± 0.07, respectively) knockdown of *ache-b* transcripts three days after exposure to virus compared to the wild type control worms (Fig 7A). No significant differences in transcription levels were observed in worms exposed to the empty vector virus (log2 = -0.05 ± 0.09). Worms in the lhp treatment group showed more reproducible knockdown efficacy than those observed in the shRNAmir treatment group. Furthermore, transduction with the lhp construct did not alter transcription levels of neuronal acetylcholinesterases 1, 2 and 3/4, while *ace-2* expression was moderately reduced following transduction with the shRNAmir cocktail. AChEB is expressed in secretory sub-ventral glands, and although accessible, we would expect a higher concentration of virus to be delivered to the intestine by pharyngeal pumping.

We next determined whether knockdown of *ache-b* transcripts persisted into adult stages of the parasite. Worms were recovered 6 days after infection with transduced L3s and expression of *ache-b* assessed again by RT-qPCR. In the first experiment, transduction of L3s with the shRNAmirs had no effect on *ache-b* transcripts in adult worms compared to wild type controls, whereas a reduction of ~20% (mean log2 ± SEM = -0.3 ± 0.08) following transduction of L3s with the lhp construct did not reach statistical significance at p<0.05 (S2A Fig). In the second experiment, no significant difference in transcription levels of *ace-b* were observed in adult worms derived from L3s exposed to viruses encoding either the lhp or the shRNAmir cocktail (S2B Fig). Thus, no clear transcript knockdown was observed in adult worms.

To further confirm functionality of virally-delivered dsRNA constructs, we investigated processing of a shRNAmir into mature siRNAs, and assessed whether a lhp could enter the siRNA pathway. Indeed, *acheb*-468 shRNAmir encoded in the 3´-UTR of the mCherry transgene was processed into mature miRNA in transduced L3s (Fig 7B), confirming successful utilisation of the miRNA pathway. Furthermore, analysis of the small RNA content of adult worms recovered from rats following transduction of L3s with lentivirus containing the *acheb* 250 bp lhp construct and subsequent infection revealed a 5-fold increase of 22G-RNAs specific to the target region, confirming successful entry into the siRNA pathway (Fig 7C). Interestingly, a 2-fold increase of target-specific 22G-RNAs was also recorded for the shRNAmir treatment group (Fig 7C). Due to the moderate increase in 22G-RNAs, we assessed different stages of the parasite for transcripts of components of the small RNAi pathway, which revealed a 70% decrease of *dcr-1*, *rde-1* and *rrf-1* in adult worms compared with activated L3s, suggesting that the capacity for RNAi-based gene silencing may decrease during development in the mammalian host (Fig 7D). Nevertheless, these data provide strong proof of principle that nematode parasites can be transduced with lentiviral vectors carrying a dsRNA expression cassette, enabling expression and processing of small RNAs to achieve transcriptional knockdown of the target gene.

## Discussion

To date, methods for genetic manipulation of animal-parasitic nematodes are extremely limited, with the notable exception of *Strongyloides* and closely related species, which can develop through one or more free-living generations outside the host, allowing the development of transgenesis techniques [25]. CRISPR-Cas9 has become an important tool for genetic manipulation of model and non-model organisms and has recently been applied to *Strongyloides stercoralis* and *Strongyloides ratti* [26,27]. Targeted mutagenesis was possible because these species exhibit a free-living stage, allowing selection of mutants in the F1 population following injection of CRISPR-Cas9 constructs into the syncytial gonad of adult females.

In another major advance, stable genomic integration of reporter genes in *Brugia malayi* was achieved by developing a co-culture system which allowed transfection of infective larvae and subsequent development to fertile adult worms following infection of gerbils. Transfection was initially performed by lipofection with *piggyBac* transposon-based plasmids [28], followed up by CRISPR-mediated targeted insertion of a secreted luciferase reporter which could be detected in a small proportion (3%) of the F1 generation microfilariae [29]. Despite this progress, major challenges remain to establish routine CRISPR-based methods to test the phenotypic effect of gene deletions, such as potential screening over multiple generations to generate homozygous mutants. Moreover, CRISPR could not be applied to genes for which the deletion mutant phenotype is lethal, or compromises infectivity and development in the mammalian host such that mutants cannot be recovered. Alternative efficient means of gene silencing therefore still have an important role in functional genomics of animal-parasitic nematodes, in particular when screening for gene products necessary for parasite survival and persistence.

We demonstrate here that VSV-G-pseudotyped lentivirus can be used to transduce *N*. *brasiliensis*. The virus was ingested by infective larvae that had been activated to feed by culture at 37°C, and taken up by intestinal cells. VSV has a remarkably robust and pantropic infectivity, mediated by its coat protein VSV-G, explained by identification of the LDL receptor and related family members as the major port of entry for VSV in mammalian cells [16]. *C*. *elegans* possesses the lipoprotein receptor proteins LRP-1 and LRP-2, which contain LDLR Class A repeats, LDLR Class B repeats, EGF-like domains and a transmembrane domain [15]. Both receptors are broadly distributed throughout the worm, with LRP-2 expressed in the intestine and multiple other tissues including those of the reproductive system [30]. *N*. *brasiliensis* L3s and adult worms express two copies of LRP-like proteins (Fig 1) as do most nematodes [15], so it is possible that these could be utilised by VSV-G-pseutotyped lentivirus to enter cells.

RNA was reverse transcribed into proviral DNA, and mCherry reporter transcripts stably produced by larvae during 4 days of in vitro culture. However, following infection of rats and subsequent recovery of adult worms, mCherry transcripts were no longer detectable. Whole genome sequencing revealed genomic integration of proviral DNA. Integration events were rare, but not too dissimilar to the integration frequency of HIV-1-derived virus in *S*. *mansoni* [31]. In *S*. *mansoni*, HIV-derived viral vectors integrate randomly into the genome [31], which may account for the low frequency of provirus detected by whole genome sequencing here, so a more directed approach such as transposon-directed insertion site sequencing [32,33] utilising lentiviral sequences may be necessary to provide detailed information on mapping integration in *N*. *brasiliensis*.

The ability to transduce *N*. *brasiliensis* with lentivirus provides another approach to address gene silencing by RNAi, which has generally proven problematic for animal-parasitic nematodes. The major problem appears to be effective delivery of sufficient dsRNA to trigger the silencing mechanism, with soaking and bacterial feeding often producing negative or inconsistent results in many species [6]. This has been circumvented by direct injection of dsRNA into the pseudocoelomic cavity of adult *Ascaris suum*, and this method of delivery, aided by the circulation of pseudocoelomic fluid, achieved successful gene knockdown across multiple tissues [34]. This is a powerful approach which allows investigation of gene function in vitro, but no opportunity to analyse roles in development and survival of parasites in the definitive host, for which sustained silencing must be initiated in infective or pre-infective larvae. This is problematic, as infective stages of many nematodes, in particular strongylids such as *N*. *brasiliensis*, exist in a state of developmental arrest which has been considered somewhat analogous to *C*. *elegans* dauer larvae [35] and do not feed. In order to overcome this problem, we cultured infective larvae at 37°C for 48 h before exposure to virus. This is sufficient to activate signalling pathways which result in resumption of feeding [17], resulting in subsequent uptake of virus (Fig 1). Inclusion of rat serum in culture medium is not necessary for activation of larvae, but is important for sustaining parasite viability such that larvae remained completely infective after in vitro manipulation (Fig 3). Single worm RT-PCR demonstrated that all activated L3s were transduced with virus (Fig 3). Interestingly, previous use of an assay based on ingestion of FITC-conjugated BSA suggested that the proportion of the larval population activated to feed over the same time period ranged between 80 and 90% [17]. We hypothesise that this discrepancy results from differing sensitivities of the experimental procedures.

This study demonstrates that shRNAmirs delivered to *N*. *brasiliensis* L3s by lentivirus can access the miRNA pathway and are processed into mature miRNAs. Furthermore, target gene knockdown of 40–50% could be achieved following delivery of shRNAmir or long hairpin constructs 3 days after exposure to virus, although there was variability in knockdown efficiency between experiments. Interestingly, *tbb-1* was susceptible to knockdown by delivery of shRNAmirs as well as lhps, whilst a moderate knockdown of *tbb-2* transcripts was only observed following exposure to lhps (Fig 6), which may be due to differences in tissue distribution of the two isoforms. In *C*. *elegans*, both *tbb-1* and *tbb-2* have a broad expression pattern, but *tbb-1* is notably present in body wall muscle, pharynx, intestine and neurons in the head, tail and ventral nerve cord, whereas *tbb-2* is highly expressed in the gonad, tail and touch receptor neurons [30,36,37]. Expression of *tbb-1* in the intestine may make this particularly susceptible to knockdown by lentiviral-delivered shRNAmirs. Independent studies from several laboratories have identified β-tubulin isotype-1 as consistently sensitive to RNAi in *Haemonchus contortus*, in contrast to many other refractory targets, and it was suggested that this may be due to high levels of expression in the posterior intestine [6]. The miRNA pathway usually does not result in systemic spread of the RNAi trigger, which is consistent with partial knockdown of *tbb-1* but not *tbb-2* by shRNAmirs. In contrast, partial knockdown of *tbb-2* by the lhp may result from systemic spread of mobile silencing dsRNAs.

Delivering small RNAs via the nematode siRNA pathway potentially offers several advantages over the miRNA pathway. Firstly, targeting itself may be more specific because the dsRNA trigger will generate several different primary siRNAs against the target. Secondly, in *C*. *elegans*, amplification of the primary RNAi trigger by RNA-dependent RNA polymerase generates secondary silencing RNAs, known as 22G-RNAs due to their length and first nucleotide [38]. Similarly, RNA polymerase-dependent secondary siRNAs have been detected in many parasitic nematodes, including *N*. *brasiliensis* [21], and these would be expected to potentiate knockdown. Thirdly, it allows for the possibility of systemic silencing beyond transduced cells via the ability of *sid-1* to transport mobile silencing dsRNAs throughout the worm [39]. Modification of the viral delivery cassette to introduce a 150–250 bp hairpin structure resulted in generation of 22G-RNAs (Fig 7C). Dicer exists in three separate complexes in *C*. *elegans*, with roles in miRNA processing, exogenous RNAi in response to external dsRNA, and endogenous RNAi in response to endogenously-generated substrates [40]. However, there is evidence that miRNAs can generate 22G-RNAs through the activity of RDE-1 [41], which may explain why there was also an increase in 22G-RNAs mapping to *ache-b* following exposure of L3s to miRNA-generating hairpins.

No obvious phenotypic effect of silencing any of the targets were observed in L3s maintained in vitro. This is most likely due to the modest knockdown achieved thus far. Whilst we might expect to observe differences in vivo following partial silencing of *tbb-1* and *tbb-2*, knockdown of *ace-b* is likely to produce more subtle effects, in particular because it is one of three genes which encode secreted AChEs with very similar properties which may function in a redundant manner [42].

Although we observed that gene silencing could be achieved by both methods described here, we would ideally aim for stronger and more sustained effects in later life cycle stages. Use of stronger promoters and insulators may lead to improved transcription of the expression cassette. Because integration of provirus into infective larvae requires active chromatin, it is conceivable that the lack of detectable mCherry transcription in adult worms could be due to chromatin-mediated transcriptional silencing of the viral integration site during parasite development. Insulators such as cHS4 have been successfully employed in *S*. *mansoni* to improve silencing effects on a retroviral vector [43]. However, recent studies have shown that most lineages within the nematode phylum have lost orthologues of many known chromatin insulator proteins, including the zinc finger protein transcriptional suppressor CTCF [44], which was lost in the common ancestor of clades III, IV and V, in which both *N*. *brasiliensis* and *C*. *elegans* are found [45]. Some of the most active promoters in *C*. *elegans* are located within CpG-rich regions enriched for the H3K4me3 modification [18]. These regions bear similarity to mammalian CpG islands, which have been successfully used to improve lentiviral delivery [46]. We therefore tested *C*. *elegans hlh11* in conjunction with *tbb2* 3´-UTR, which led to improved transcription of the transgene. However, sustained expression throughout the life cycle was still not achieved. A proportion of provirus could remain in an unintegrated episomal state, which could lead to loss of the expression cassette altogether in cell division during worm development. A similar situation was observed in earlier attempts at plasmid-based transgenesis in *Strongyloides stercoralis*, in which constructs were configured with a *Strongyloides*-specific promoter and a *C*. *elegans* 3´-UTR, resulting in developmental silencing of expression, but substitution of the latter regulatory element with a *Stronglyloides*-specific 3´-UTR resulted in sustained expression of the transgene throughout subsequent life cycle stages [47].

The current study demonstrates that viral transduction of a model nematode parasite is feasible, and that this can be utilised to genetically manipulate the organism via expression of exogenous genes and induction of gene silencing. Detection of lentiviral sequences in the F1 generation raises the possibility of manipulating gene expression in multiple tissues and generating stable transgenic parasite lines. This was surprising, as it is unclear how lentivirus could access the germline in L3s. The lentiviral transfer plasmids used contain a deletion in the U3 region of the 3' LTR (SIN-LTR) which renders the virus replication-incompetent. Infection of germ cells cannot therefore be a secondary event following initial infection of intestinal epithelial cells but must occur directly, accessed from the external environment by lentivirus. The failure to detect lentiviral sequences in F1 L3s by single worm PCR on a limited number of larvae suggests that entry into the germline may be a rare event. It might be possible to select transgenic worms via fluorescent reporters, but this would require further optimisation of the expression cassette to overcome the problem of transcriptional silencing during development observed in this study. We are therefore working to refine the system, in particular by identification of suitable *N*. *brasiliensis*-specific regulatory elements in order to promote sustained expression of transgenes throughout the life cycle.

## Materials and methods

### Ethics statement

This study was approved by the Animal Welfare Ethical Review Board at Imperial College London and was licensed by and performed under the UK Home Office Animals (Scientific Procedures) Act Personal Project Licence number PFA8EC7B7: ‘Immunity and immunomodulation in helminth infection’.

### Vector construction

The pGIPZ lentiviral vector used previously [9] was modified as follows: the coding sequence for fluorescent protein mCherry was optimised for *N*. *brasiliensis* codon usage (*Nb-mCherry*) and synthesized (GeneArt, Thermo Fisher Scientific) for subsequent integration via *Spe*I and *Not*I restriction sites. The *C*. *elegans* hlh11 promoter [18] and *tbb-2* 3´-UTR [20] were amplified by single worm PCR. In brief, one adult *C*. *elegans* was digested in 10 μl 1x OneTaq standard PCR buffer (New England Bioloabs, NEB) supplemented with 1 mg ml^-1^ proteinase K (Sigma) for 90 min at 60°C. Gene-specific regions were then amplified in 50 μl reactions using OneTaq polymerase (NEB) according to manufacturer's recommendations with 1 μl of the worm digest mixture serving as template and 250 nM of the following primers (lower case indicating nucleotides added for cloning purposes and restriction site underlined): *hlh-11* promoter sense: 5´-atacgctagcGACCCCGTCGGTGCCCATCC-3´, antisense: 5’-agatactagtCGGTGGTGGTGGTGGACTGATCG-3’; *tbb-2* 3´-UTR sense: 5’-atccacgcgtATGCAAAATCCTTTCAAGCATTC-3’, antisense: 5´-agatggcgcgccGGTACC GACTTTTTTCTTGGCGGCACAATAAAG-3´.

For miRNA-adapted shRNA design (shRNAmir), target gene-specific hairpin stem sequences were inferred using the algorithm DSIR [48] and cloned into pGIPZ plasmids for expression as primary transcripts of human miRNA30, as previously described [9]. The sequences for the mature artificial miRNAs are listed in Table 1. For long hairpin designs, target gene regions of 145 to 186 consecutive nucleotides that contained the DSIR-predicted siRNA binding sites were chosen. For improved vector stability, loop sequences of 64 or 55 nucleotides were introduced for *tbb-1* and *tbb-2* hairpins, respectively. Sense and antisense sequences for *tbb-1* were amplified from *N*. *brasiliensis* cDNA by conventional PCR using OneTaq polymerase (NEB) and synthesized for *tbb-2* (GeneArt, Thermo Fisher Scientific) for subsequent integration into the target vector via *Spe*I and *Bam*HI (sense) or *Bam*HI and *Mlu*I (antisense) restriction sites. Sense and antisense sequences for *ache-B* synthesized (GeneArt, Thermo Fisher Scientific) for subsequent integration into the target vector via *Spe*I and *Xho*I (sense) or *Xho*I and *Mlu*I (antisense) restriction sites. Virus-encoding plasmids were maintained in NEB stable *Escherichia coli* (NEB). Positive transformants were selected on LB agar plates containing 25 μg ml^-1^ zeocin (Life Technologies) and 100 μg ml^-1^ ampicillin (Sigma). Constructs were verified for error-free integration of transgenes by routine Sanger sequencing (Eurofins Genomics).

### Virus production

VSV-G-pseudotyped, replication-incompetent virus particles were produced in HEK293T cells maintained in Dulbecco’s Modified Eagle’s Medium (DMEM) at 37°C, 10% foetal calf serum (FCS), 2 mM L-glutamine, 100 units ml^-1^ penicillin and 100 μg ml^-1^ streptomycin (all Sigma). In brief, per well of a 6-well plate, 2.5 x10^6^ cells were transfected with 2.5 μg of virus-encoding plasmid, 2.5 μg of psPAX2 and 1 μg of pMD2.G using Lipofectamine 2000 at a ratio of 1:2.5 (Life Technologies). PsPAX2 and pMD2.G were a gift from Didier Trono (Addgene plasmids # 12260 and 12259, respectively). After 16 hrs, the transfection medium was replaced with 2 ml of reduced serum culture medium (5% FCS) supplemented with 20 mM HEPES (Sigma) and 10 μM cholesterol (balanced with methyl-β-cyclodextrin, Sigma) [49]. After an additional incubation of 48 hrs at 37°C and 10% CO_2_, the virus-containing cell supernatant was harvested, centrifuged at 1,000 x *g* for 10 min at 4°C, passed through a 0.45 μm Acrodisc syringe filter (Pall) and stored at -80°C as 1 ml aliquots. Functional virus titres were estimated by RT-qPCR following DNAseI treatment (Sigma) and reverse transcription using Superscript IV and oligo(dT)20 primers (both Life Technologies) as previously described [9].

For analysis of virus uptake, 5 μl of Vybrant DiD (Life Technologies) was added to 1 ml of virus-containing supernatant for 20 min at 22°C. Virus particles were then pelleted by centrifugation over a sucrose gradient at 10,000 x *g* for 4 hrs at 4°C as previously described [50] and resuspended in cold PBS. DiD-labelled virus incubated at 95°C for 15 min served as a heat-inactivated virus control.

### Parasite infection, recovery and transduction with virus

*N*. *brasiliensis* were maintained in male SD rats, and infective larvae isolated from faecal cultures using a Baermann apparatus and washed extensively in worm wash buffer (PBS, 0.45% glucose, 100 units ml^-1^ penicillin, 100 μg ml^-1^ streptomycin, 100 μg ml^-1^ gentamicin) with centrifugation at 150 x *g* for 1 min between washes. Larvae were activated to feed as previously described [17] for 48 to 72 hrs in RPMI1640, 0.65% glucose, 2 mM L-glutamine, 100 U ml^-1^ penicillin, 100 μg ml^-1^ streptomycin, 100 μg ml^-1^ gentamicin, 20 mM HEPES, 2% rat serum (worm culture medium), then washed twice in serum-free medium prior to exposure to lentivirus. Approximately 10,000 activated L3s were exposed to virus at various MOIs in 1 ml of serum-free medium containing 10 μg ml^-1^ polybrene (Sigma). Controls included unexposed (wild type) worms and worms exposed to virus lacking a small hairpin structure (empty vector). Virus-containing culture medium was replaced with worm culture medium after 18 to 24 hrs and larvae incubated for a further 48 hrs before analysis for transgene expression and knockdown.

### Recovery of transgenic worms from the rodent host

Activated L3s were exposed to virus for 18 hrs and washed three times in worm wash buffer. Male SD rats were infected with approximately 4,000 L3s, and adult worms recovered from the intestines at 5 to 7 days post-infection. Worms were washed, pelleted and frozen for isolation of genomic DNA or stored in TriReagent (Sigma) for subsequent RNA extraction.

### ATP assay

To assess parasite viability, 100 L3s were homogenised in 100 μl of PBS, and ATP content determined using the CellTiter-Glo luminescent cell viability assay [51] according to manufacturer's instructions (Promega).

### Confocal microscopy

Parasites were washed in PBS, fixed in 10% neutral-buffered formalin for 10 min on ice, washed in PBS and mounted in Vectashield mounting medium (Vector Labs). Imaging was performed with a Zeiss 510 META and images were analysed using FUJI software.

### Reverse transcription PCR (RT-PCR) and real-time quantitative PCR (RT-qPCR)

Total RNA was extracted using TRIreagent (Sigma) and converted to cDNA using iScript cDNA kit (Biorad) following the removal of contaminating genomic DNA by DNAseI (Sigma) treatment. RT-PCR was carried out using OneTaq DNA polymerase (NEB) according to the manufacturer’s recommendations. RT-qPCR was carried out with a Step-One PLUS Fast Real-time PCR cycler (Applied Biosystems) under standard fast cycling conditions using PowerUP SYBR Green PCR Master Mix (Applied Biosystems) and 250 nM of target gene specific forward and reverse primers. PCR amplification efficiencies were established for each primer pair [52] and ranged between 1.9 and 2.1. A panel of genes [53] was analysed for suitable reference genes using qbasePLUS software (Biogazelle) [54]. Cycle threshold (Ct) values of target genes were normalised to the geometric mean of *eif-3C*, *idhg-1* and *rbd-1* and calibrated to the mean untreated control (wild type) samples for relative quantification by the comparative Ct method [55]. The primers were:

*ace-1* sense: 5’*-*CGTGCACGGATACGAGATCA-3’, antisense 5’-GTTCGAGCAAAATTCGCCCA-3’; *ace-2* sense: 5’-ACTCACCACTCGTCGAACAC-3’, antisense 5’-ATAGTCGCCAAGGAATCGCC-3’; *ace-3* sense: 5’-ACGCAGTTCTATGCCGAACA-3’, antisense: 5’-ATTCGCGCTAGACGGTTGAT-3’; *dcr-1* sense: 5’-GCCTTATGTGGTCCCGTCAA-3’, antisense: 5’-GTGGTACCAGACGGGTGAAG-3’; eif*-3C* sense: 5´-GAACACGTTGTAGCTGCGTCA-3´, antisense: 5´-AATAGGTTCTCAGCGATTCCGTT-3´; *idhg-1* sense: 5´-CAGAAATTGGGAGACGGCCT-3´, antisense: 5´-CCGAGAAACCAGCTGCATAGA-3´; *lrp-1* sense: 5´-GGTTACTCCTGCGGTTGTCC-3´, antisense: 5´-AACGCCATTACCTCTGTTCGT-3´; *lrp-2A* sense: 5´-GGAGTTCTGCGGTTATGAGCA-3´, antisense: 5´-TCTGAGAGGCTTCGCAAGTTT-3´; *lrp-2B* sense: 5´-GGAAGTGCGACTCCGACAAT-3´, antisense: TTGCACAGGAGAATTGATTGACA-3´; Nb-mCherry sense: 5´-GACGGACCAGTGATGCAGAA-3´, antisense: 5´-ACGTTGTAAGCACCAGGGAG-3´; *rde-1* sense: 5’-CCGAGCCAAGTCGTTGATCT-3’, antisense: CATGACCGCATCTTTCGCAG-3’; *rbd-1* sense: 5´-TCTCCCTGGGGCAGAAAAAC-3´, antisense: 5´-TCCAAGAATGAGATTTCACAGCA-3´; *rrf-1* sense: 5’-GGTCTGATCCTGCCATCTCG-3’, antisense: 5’-TGACGTTCGGGTCTTTGAGG-3’; *sid-1* sense: 5´-TCGCGGAGTGCACAATAACT-3´, antisense: 5´- CGATGAACGGCGTATCCAG-3´; *tuba* sense: 5´-TCGTACCGTACCCTCGCAT-3´, antisense: 5´-GATCGCATTTCACCATCTGATT-3´; *tbb-1* sense: 5´-CAACCATGCGTGAGATCGTG-3´, antisense: 5´-TCGCCCTTGTAGGTTCCATC-3´; *tbb-2* sense: 5´-TCACCCAGCAGATGTTCGAC-3´, antisense: 5´-TCACGTTGTTCGGGATCCATT-3´.

### Single worm PCR and RT-PCR

One L3 was digested in 1 μl of 1x OneTaq standard PCR buffer (NEB) supplemented with 1 mg ml^-1^ proteinase K (NEB) for 30 min at 55°C and subsequent inactivation of proteinase K by incubation at 95°C for 10 min. Worm digest mixtures were diluted to 20 μl with nuclease-free water or subjected to cDNA synthesis using Maxima H Minus cDNA synthesis kit (Thermo Fisher) as described previously [56]. The resulting cDNA was diluted to 20 μl with nuclease-free water. Gene-specific regions were then amplified in 20 μl reactions using Quantitect (Qiagen) or Q5 polymerase (NEB) according to manufacturer's recommendations with 2 μl of the worm digest mixture or cDNA serving as template, and 500 nM primers (as above). The presence of mCherry was assessed using the following primers: Nb-mCherry II sense: 5´-ACGAACTTCCCGAGCGACGGACCA-3´, antisense: 5´-CGCCCTTGAGAGCGCCGTCC-3.

### Detection of proviral DNA

For integration studies in L3s, genomic DNA was isolated from transduced worms at 48 and 72 hrs post exposure to virus using the DNeasy Blood and Tissue DNA extraction kit (Qiagen). Proviral DNA was detected by conventional PCR using OneTaq polymerase and primers spanning the transgene expression cassette resulting in a 1.4 kb amplicon (CMV forward 5´-TGGGACTTTCCTACTTGGCAG-3´, miR30 reverse 5´-TTCCAGACGCGTCCTAGGTAATACGACTCAC-3´). For detection of residual plasmid DNA in gDNA preparations, primers binding the WPRE and SV40 promoter region were used to amplify a 1.8 kb fragment (WPRE forward 5´-CAATTCCGTGGTGTTGTCG-3´, SV40P reverse 5´-AATSGCTCAGAGGCAGAGGC-3´). Amplification of lentivirus-encoding plasmid DNA served as a positive control. For detection of episomal proviral DNA, nested PCR was performed using the 2x Q5 Polymerase master mix (NEB) in 25 μl reactions with 300 ng of gDNA in the first PCR step and 10% of the resulting fragments in a successive PCR and the following primers: Nested 1 (sense 5´-TAGAAGCACAAGAGGAGGAGGAGGTGG-3´, antisense 5´-GCAAGCCGAGTCCTGCGTCG-3´); Nested 2 (sense 5´-TAAGACCAATGACTTACAAGGCAGCTGTAGATCTTAG-3´, antisense 5´-CCCTTTCGCTTTCAAGTCCCTGTTCGGGCG-3´). Amplification of the 1-LTR was confirmed by routine Sanger sequencing.

### Heritable transgenesis

Rats were infected with 4,000 transduced or wild type L3s and faeces collected on day 7 to day 8 post infection for establishment of faecal cultures and recovery of F1 L3. Adult worms were recovered from infected rats at day 8 post infection. All worms recovered from one individual rat were incubated overnight at 37°C, 5% CO_2_ in 20 ml of worm culture medium. F1 eggs were recovered from culture medium by passage through a 100 μm cell strainer and subsequent centrifugation at 1000 x *g* for 10 min. The resultant egg pellet (10 μl packed volume) and a similar volume of recovered L3s (~2000 worms) were resuspended in 100 μl of digestion buffer (1x OneTaq buffer supplemented with 1 mg ml^-1^ proteinase K, NEB) and incubated for 30 min at 55°C. Proteinase K was inactivated by incubation at 95°C for 10 min. F1 worm and egg extracts were then assessed for the presence of proviral DNA by PCR using the 2x Q5 Polymerase master mix (NEB) in 25 μl reactions with 3 μl of worm extract serving as a template and primers as described above.

### Analysis of integration events

Library construction and high-throughput sequencing of paired-end 150 bp libraries was performed by Eurofins Genomics. To identify potential integration events, reads were first mapped to a combined genome built from the *N*. *brasiliensis* draft genome (Wormbase Parasite V5) and lentiviral DNA using bowtie2-build. Reads that did not align to these sequences were selected as potential split reads aligning to both lentiviral DNA and the *N*. *brasiliensis* genome across the integration site. These reads were then split into sub-reads of varying lengths using a custom Perl script and the sub-reads were aligned separately to *N*. *brasiliensis* and lentiviral DNA. Reads that contained at least one sub-read that aligned to *N*. *brasiliensis* and at least one sub-read that aligned to lentiviral DNA were selected. To examine whether the number of reads identified as aligning to lentivirus was significantly greater than DNA from control (uninfected) worms, sequencing data from the infected worms was downsampled by randomly selecting the number of reads obtained from the control worms, as the control library generated a smaller number of reads in total. The downsampled reads were then aligned to lentiviral DNA and the number of aligned reads quantified. This process was repeated 100 times to generate a distribution, and this was compared to the number of reads from control worms that aligned to lentiviral DNA.

### Processing of shRNAmir and long hairpin dsRNA

Total RNA was isolated from transduced worms at 48 and 72 hrs post exposure to virus using TriReagent and analysed for presence of mature miRNAs generated from shRNAmir_468 using the miScript detection system (Qiagen) and a miRNA-specific forward primer following the manufacturer’s instructions.

For detection of 22G-RNAs, 1–5 μg of total RNA was treated with 7.5 U of RNA 5´ pyrophosphohydrolase (RppH, NEB) for 1 hr at 37°C in 20 μl reactions to convert 5´-di/triphosphates to monophosphate. RNA was then purified by phenol/chloroform extraction and resuspended in ultrapure water. Small RNA libraries were prepared using the Truseq Small RNA library prep kit (Illumina) following the manufacturer’s instructions and purified by ethanol precipitation after separation on a 6% polyacrylamide gel. Purity and size of extracted libraries was confirmed using a tapestation (Agilent). Libraries were sequenced to generate 50 bp single end reads by the MRC London Institute of Medical Sciences sequencing facility.

### Small RNA sequencing data analysis

High-throughput sequencing reads were processed to remove adapters using fastq-clipper and short reads <15 nucleotides in length were discarded. Reads were then aligned to the *N*. *brasiliensis acheB* sequence (WBPS5). In *N*. *brasiliensis*, secondary small RNAs are predominantly 23 nucleotides long and start with a G [21]. Reads matching these characteristics were selected and the number mapping to *acheB* quantified. Data were normalised to the total number of reads.

### Statistics

Treatment groups were analysed for significant differences with the Kruskal-Wallis test and Dunns *post-hoc* test in relation to the wild type or empty vector control group. Values are expressed as the median with range or the mean ± SEM, and significant differences were determined using GraphPad Prism. P values of <0.05 were considered significant. *p<0.05, **p<0.01, ***p<0.001.

### Accession numbers

WormBase ParaSite or GenBank accession numbers of genes used in this study are provided in S1 Table.

## Supporting information

S1 Table WormBase ParaSite or GenBank accession numbers of genes used in this study. (DOCX)

S1 Fig Integration of lentivirus into *N*. *brasiliensis* genome. (A) Histogram demonstrating significantly more reads aligning to lentivirus from infected worms compared to control uninfected worms. The distribution of detected reads in 100 random samples of sequences from infected worms is shown compared to the number of detected reads in the control sample (0). (B) Split reads aligning to both viral DNA and *N*. *brasiliensis* genomic DNA.
(TIF)

S2 Fig Lack of reproducible transcriptional knockdown of secreted *acetylcholinesterase B* in adult worms. L3s were exposed to a cocktail consisting of two shRNAmir lentiviruses (Table 1) or with lentivirus encoding a 250 bp lhp targeting *ache-b*. Control worms were left untreated (wild type, WT) or transduced with virus encoding mCherry lacking a hairpin sequence (empty vector, EV). Rats were infected with transduced or control L3, adult worms removed 6 days post-infection and transcripts assessed by RT-qPCR relative to wild type control worms and normalised against the geometric mean of Ct values of reference genes *eif-3C* and *idhg-1*. Box plot representing the median and upper/lower quartile of data from 3–4 biological replicates consisting of ~500 worms each. Whiskers indicate the highest or lowest value. (A) Experiment 1. (B) Experiment 2. Treatment groups were analysed for significant differences with the Kruskal-Wallis test and Dunns *post-hoc* test in relation to the wild type or empty vector control group.
(TIF)
