# Supplementary material for: Lentiviral transduction facilitates RNA interference in the nematode parasite Nippostrongylus brasiliensis
Source: PLoS Pathog. 2021 Jan 26;17(1):e1009286. doi: 10.1371/journal.ppat.1009286 (PMC7864396; doi:10.1371/journal.ppat.1009286)
Supplement: S1 Table — (DOCX) [file ppat.1009286.s001.docx]

**S1 Table. WormBase ParaSite or GenBank accession numbers of genes used in this study.**

| Gene symbol | Gene name | Accession number |
| --- | --- | --- |
| *ace-1* | Acetylcholinesterase 1 | NBR_0000665001 |
| *ace-2* | Acetylcholinesterase 2 | NBR_0000669601 |
| *ace-3/4* | Acetylcholinesterase 3/4 | NBR_0000102801 |
| *ache-b* | Acetylcholinesterase B | AF052508.1 |
| *dcr-1* | Endoribonuclease dcr-1 Death-promoting deoxyribonuclease | [NBR_0000904701](https://parasite.wormbase.org/Nippostrongylus_brasiliensis_prjeb511/Gene/Summary?db=core;g=NBR_0000904701) |
| *eif-3c* | Eukaryotic translation initiation factor 3 | NBR_0001150401 |
| *idhg-1* | Isocitrate dehydrogenase subunit | NBR_0000658601 |
| *lrp-1* | Low-density lipoprotein receptor-related  protein 1 | NBR_0002125301 |
| *lrp-2* | Low-density lipoprotein receptor-related  protein 2 | NBR_0000573301 |
| *rbd-1* | RNA binding domain protein | NBR_0000030901 |
| *rde-1* | RNA interference promoting factor | [NBR_0001440701](https://parasite.wormbase.org/Nippostrongylus_brasiliensis_prjeb511/Gene/Summary?g=NBR_0001440701) |
| *rrf-1* | RNA-dependent RNA polymerase Family | \|  \| [NBR_0001395701](https://parasite.wormbase.org/Nippostrongylus_brasiliensis_prjeb511/Gene/Summary?db=core;g=NBR_0001395701) \| \| --- \| --- \| |
| *sid-1* | Systemic RNA interference defective | NBR_0001626601 |
| *tbb-1* | Tubulin beta isoform 1 | NBR_0000799801 |
| *tbb-2* | Tubulin beta isoform 2 | NBR_0000856601 |
| *tuba* | Tubulin alpha chain | [NBR_0001606301](https://parasite.wormbase.org/Nippostrongylus_brasiliensis_prjeb511/Gene/Summary?g=NBR_0001606301;r=NBR_scaffold0002268:126-7580) |
|  |  |  |
