# Supplementary figures and images for: Lentiviral transduction facilitates RNA interference in the nematode parasite Nippostrongylus brasiliensis
Source: PLoS Pathog. 2021 Jan 26;17(1):e1009286. doi: 10.1371/journal.ppat.1009286 (PMC7864396; doi:10.1371/journal.ppat.1009286)

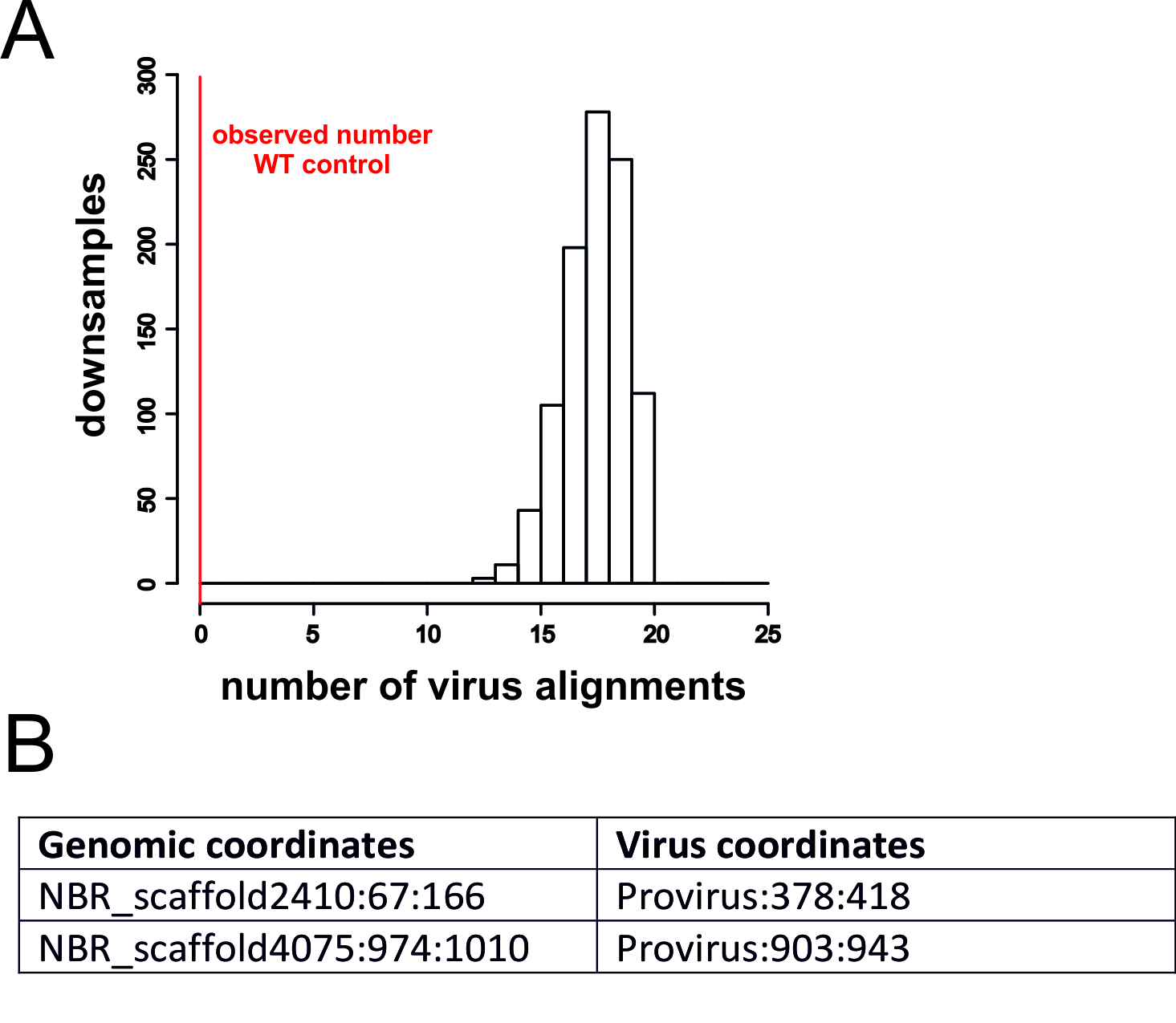

Supplement: S1 Fig — (A) Histogram demonstrating significantly more reads aligning to lentivirus from infected worms compared to control uninfected worms. The distribution of detected reads in 100 random samples of sequences from infected worms is shown compared to the number of detected reads in the control sample (0). (B) Split reads aligning to both viral DNA and N. brasiliensis genomic DNA. (TIF) [file ppat.1009286.s002.tif]

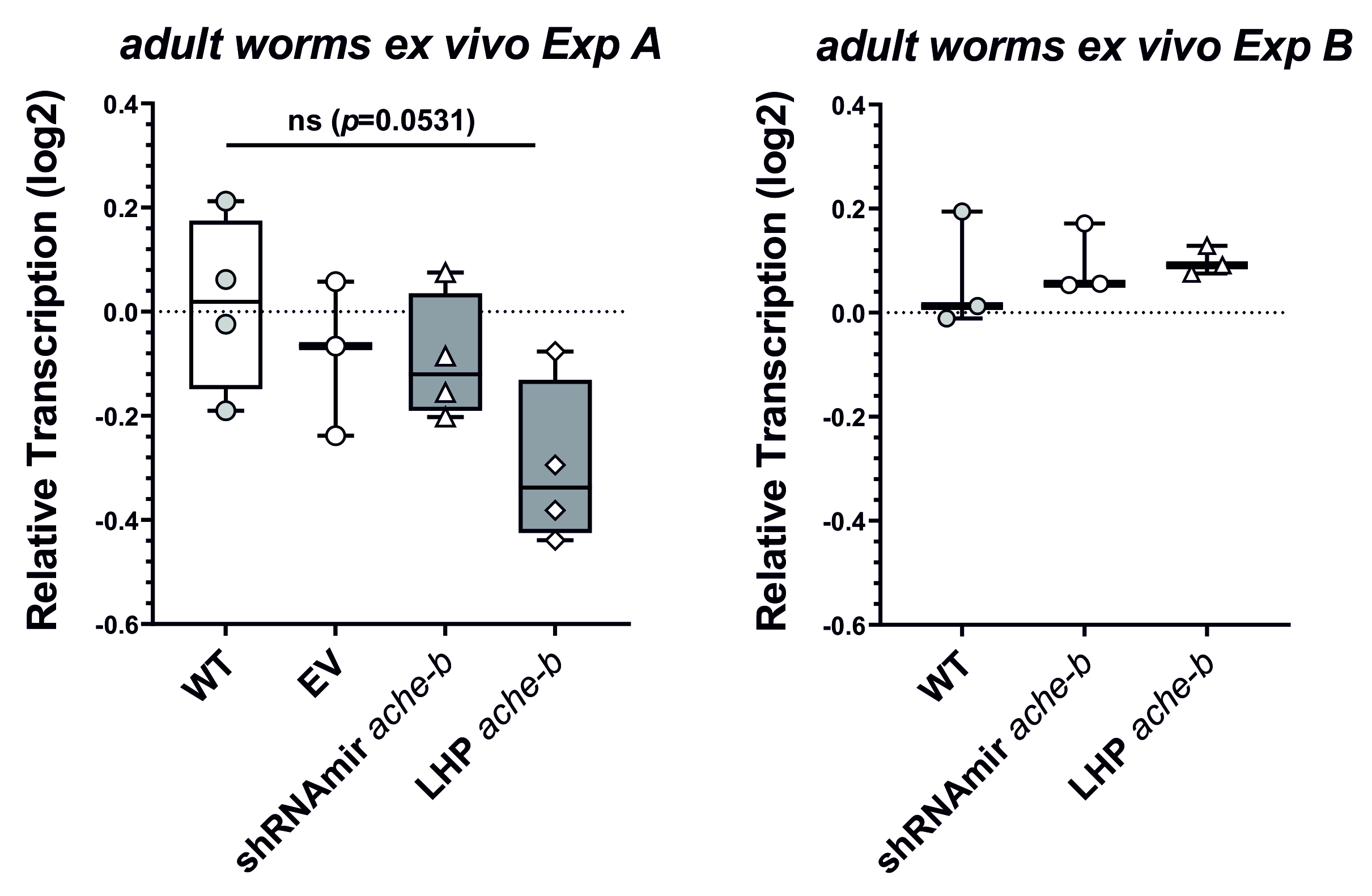

Supplement: S2 Fig — L3s were exposed to a cocktail consisting of two shRNAmir lentiviruses (Table 1) or with lentivirus encoding a 250 bp lhp targeting ache-b. Control worms were left untreated (wild type, WT) or transduced with virus encoding mCherry lacking a hairpin sequence (empty vector, EV). Rats were infected with transduced or control L3, adult worms removed 6 days post-infection and transcripts assessed by RT-qPCR relative to wild type control worms and normalised against the geometric mean of Ct values of reference genes eif-3C and idhg-1. Box plot representing the median and upper/lower quartile of data from 3–4 biological replicates consisting of ~500 worms each. Whiskers indicate the highest or lowest value. (A) Experiment 1. (B) Experiment 2. Treatment groups were analysed for significant differences with the Kruskal-Wallis test and Dunns post-hoc test in relation to the wild type or empty vector control group. (TIF) [file ppat.1009286.s003.tif]
